# Demyelinating Diseases: From Molecular Mechanisms to Therapeutic Strategies

**DOI:** 10.3390/ijms24054596

**Published:** 2023-02-27

**Authors:** Antonietta Bernardo, Sergio Visentin

**Affiliations:** National Center for Drug Research and Evaluation, Istituto Superiore di Sanità, Viale Regina Elena, 299, 00161 Rome, Italy

Demyelinating diseases are a group of pathologies characterized by the alteration of myelin—that is, the coating that wraps around most of the nerve fibres of the central and peripheral nervous system, whose goal is the improvement of nerve conduction and the preservation of energy spent during action potential propagation. They are very disabling diseases, affecting millions of people worldwide. Different types of demyelinating disorders are characterized by peculiar features that allow their classification. According to their pathogenesis, the wide variety of demyelinating diseases are classified into the following categories: due to immune-mediated inflammatory processes, infectious diseases caused by metabolic disorders, and hypoxic–ischaemic forms. The myelin produced by oligodendrocytes in the central nervous system (CNS) differs, in terms of chemical and immunological features, from that formed by Schwann cells in the peripheral nervous system (PNS). Accordingly, some demyelinating diseases mainly affect the peripheral nerves, while others primarily affect the CNS. In most cases, however, no effective pharmacological treatment can completely restore the standard functionality of myelin and nerve conduction. The purpose of this Special Issue, entitled “Demyelinating Diseases: From Molecular Mechanisms to Therapeutic Strategies”, published in the *International Journal of Molecular Sciences* [1], was to gather reviews and original research articles focused on the identification and preclinical validation of targets for therapeutic approaches to be utilized in preventive or curing strategies, in order to improve the quality of life of patients suffering from these severe pathologies.

The topics ranged from identifying the cellular and molecular mechanisms activated in some pathologies linked to demyelination processes to identifying any drugs capable of counteracting the highlighted phenomena.

In one of the three reviews presented, Balestri et al. [2] show an exciting overview of how in multiple in vitro models, the reactivation of their stem cell niche can promote oligodendrocyte maturation. Changes in certain factors and understanding the activation mechanisms could help regulate the ability of OPC and OL to remyelinate axons and possibly restore the adult brain after myelin injury. The constructed models could be exploited in this context to screen new CNS regenerative drugs and discover new agents promoting remyelination.

The review presented by Kim [3] discusses the innate and adaptive immune responses to Theiler’s murine encephalomyelitis virus (TMEV), which appears to be involved in the initial infliction of tissue damage evident in multiple sclerosis (MS). The identification of cytokines (such as IL-6, IL-1β, and/or IL-17) produced by cell populations permissive to infection, such as professional antigen-presenting cells (dendritic cells, macrophages, and B cells) and non-professional (glial cells), and the mechanisms activated by these molecules, could favour the understanding of the critical points capable of developing pathogenic T cell responses and viral persistence. The functional inhibition of these crucial inflammatory cytokines may prevent the pathogenesis of TMEV-induced demyelinating disease.

The last review by Khodanovich and colleagues [4] underlines the importance of using more innovative techniques to combine a quantitative MRI assessment of myelin with neurological and psychological studies further to understand post-COVID-19 mechanisms and associated complications with demyelination. Current knowledge on neurological sequelae in post-COVID-19 patients manifesting neurological complications and cognitive impairment would favour cerebral demyelination as a possible mechanism of these complications, as evidenced by neuroimaging methods. The authors underline how, in most clinical studies, in post-COVID-19 patients, the use of conventional MRI techniques may not be sufficient to give a detailed picture of the actual conditions, revealing only large lesions but not subtle changes invisible to the eye.

Of the four original research papers presented, two are in vitro, and two are in vivo. In the in vitro studies, Dimethyl fumarate (DMF) [5] and Telmisartan (TLM) [6]—both already in clinical use—could act with anti-inflammatory and antioxidant effects on Oli neu cells, respectively, or promote the myelination of highly purified oligodendrocyte cells of rat, even after insults.

DMF has been approved as the first oral first-line therapy for relapsing–remitting multiple sclerosis (RRMS), due to its immunomodulatory and neuroprotective effects and favourable risk–benefit profile. The authors [5] also confirm the anti-inflammatory effect in Oli neu cells, counteracting the production of IL-6 and TNFα after stimulation with LPS. DMF can also protect OPCs from oxidative stress and slow cell proliferation in favour of differentiation into pro-myelinating OLs.

TLM is an antihypertensive drug commonly used to treat cardiovascular disorders, metabolic syndrome, and kidney disease. In addition to acting as the angiotensin II receptor blocker, TLM modulates the PPAR-γ nuclear receptor, whose activation promotes OPC protection and differentiation. The authors [6] demonstrated that TLM, through a PPAR-γ mediated mechanism, promotes OP differentiation under physiological and pathological conditions, such as those mimicking the accumulation of intracellular cholesterol and morphological alterations typical of Niemann-Pick disease.

In an in vivo study, Seo et al. [7] showed, in a model of Tamalin knockout (KO) zebrafish and mice, that Tamalin (a post-synaptic scaffolding protein that plays an essential role in synaptic plasticity in vitro by controlling the ligand-dependent trafficking of group 1 mGluRs) can play a prominent role in neuronal and oligodendrocyte survival and myelination through the regulation of mGluR5 in the CNS, verifying any activated mechanisms.

In another in vivo model, Mihai et al. [8] proved the effects of three drugs, venlafaxine (antidepressant, serotonin-norepinephrine reuptake inhibitor); risperidone (atypical antipsychotic); and febuxostat (gout medication, xanthine oxidase inhibitor) in a cuprizone mouse model of acute demyelination, hypothesizing an antagonistic effect on TRPA1 (transient receptor potential ankyrin 1). All three drugs considered could be used to treat demyelinating diseases after verifying the beneficial effects observed in behaviour tests, and myelin integrity and different biochemical markers after cuprizone intoxication.

Overall, the seven contributions published in this Special Issue illustrate how with different mechanisms, drugs already in use in the clinic can be used for other pathologies different from the original medical indications. Insights into the mechanisms activated and identifying new therapies for the numerous demyelinating diseases could be relevant for public health protection.

## References

[1] Special Issue “Demyelinating Diseases: From Molecular Mechanisms to Therapeutic Strategies”. Int. J. Mol. Sci..

[2] Balestri S., Del Giovane A., Sposato C., Ferrarelli M., Ragnini-Wilson A. (2021). The Current Challenges for Drug Discovery in CNS Remyelination. Int. J. Mol. Sci..

[3] Kim B.S. (2021). Excessive Innate Immunity Steers Pathogenic Adaptive Immunity in the Development of Theiler’s Virus-Induced Demyelinating Disease. Int. J. Mol. Sci..

[4] Khodanovich M.Y., Kamaeva D.A., Naumova A.V. (2022). Role of Demyelination in the Persistence of Neurological and Mental Impairments after COVID-19. Int. J. Mol. Sci..

[5] Guerriero C., Puliatti G., Di Marino T., Tata A.M. (2022). Effects Mediated by Dimethyl Fumarate on In Vitro Oligodendrocytes: Implications in Multiple Sclerosis. Int. J. Mol. Sci..

[6] Bernardo A., Malara M., Bertuccini L., De Nuccio C., Visentin S., Minghetti L. (2021). The Antihypertensive Drug Telmisartan Protects Oligodendrocytes from Cholesterol Accumulation and Promotes Differentiation by a PPAR-γ-Mediated Mechanism. Int. J. Mol. Sci..

[7] Seo Y., Mo S., Kim S., Kim H., Park H.-C. (2022). Tamalin Function Is Required for the Survival of Neurons and Oligodendrocytes in the CNS. Int. J. Mol. Sci..

[8] Mihai D.P., Ungurianu A., Ciotu C.I., Fischer M.J.M., Olaru O.T., Nitulescu G.M., Andrei C., Zbarcea C.E., Zanfirescu A., Seremet O.C. (2021). Effects of Venlafaxine, Risperidone and Febuxostat on Cuprizone-Induced Demyelination, Behavioral Deficits and Oxidative Stress. Int. J. Mol. Sci..

